# Addressing negative symptoms of schizophrenia in a Psychosis Day Hospital: a case report

**DOI:** 10.1192/j.eurpsy.2024.1517

**Published:** 2024-08-27

**Authors:** F. Mayor Sanabria, E. M. Fernández Fonollosa, C. M. Gil Sánchez, A. Fuentes Merlos, E. X. González Vivero, S. Puyal González, M. Fernández Fariña, B. Serván Rendon-Luna

**Affiliations:** ^1^Instituto de Psiquiatría y Salud Mental, Hospital Clínico San Carlos, Madrid; ^2^Hospital de La Línea, La Línea de La Concepción, Spain

## Abstract

**Introduction:**

Negative symptoms are present in more than two thirds of schizophrenic patients throughout the evolution of the disorder. These include symptoms related to reduced motivation or pleasure, such as avolition, anhedonia and asociality, and reduced expressivity, including alogia and blunted affect.

We present the case of a 24-year-old man who was admitted to our Psychosis Day Hospital after several psychotic episodes, presenting with prominent negative symptomatology that was imbued with mystical delusional beliefs.

**Objectives:**

1. To describe the clinical particularities of this case, focusing on the improvement of negative symptoms during the course of treatment at our Day Hospital.
2. To review the available evidence regarding the pharmacological and psychotherapeutic management of negative symptoms of schizophrenia.

**Methods:**

A review of the patient’s clinical history and complementary tests were carried out. Likewise, we reviewed the available literature in relation to the management of negative symptoms of schizophrenia in an ambulatory setting.

**Results:**

The patient was admitted to our Day Hospital after four psychiatric hospitalizations due to mystical delusions, ideas of grandiosity and hyper-spirituality, along with prominent negative symptoms at the moment of inclusion at our centre, including social withdrawal, diminished affective response, lack of interest in the academic sphere and poor social drive. Although previous positive symptoms were present in a lesser degree, the patient interpreted the presence of the negative symptoms described above as a “punishment” or “test” from spiritual creatures.

Management of negative symptoms represents a major unmet need in schizophrenia. Modest effect size evidence for pharmacological approaches favours the use of antipsychotic in monotherapy and augmentation of antipsychotic treatment with other agents, such as antidepressants. Scarce evidence regarding psychotherapeutic approaches to these symptoms points to the use of cognitive behaviour therapy and social skills training.

**Conclusions:**

- Clinical identification and characterization of negative symptoms is crucial when treating patients with schizophrenia, as these are associated with important disability and poorer functional outcomes.
- Differentiation of primary and secondary negative symptoms is a key aspect in the evaluation and management of schizophrenic patients.
- This case outlines the coexistence of positive and negative symptoms, and illustrates the challenges in the pharmacological and psychotherapeutic management of these symptoms at a Psychosis Day Hospital.

**Disclosure of Interest:**

None Declared

